# A Machine Learning Approach with Human-AI Collaboration for Automated Classification of Patient Safety Event Reports: Algorithm Development and Validation Study

**DOI:** 10.2196/53378

**Published:** 2024-01-25

**Authors:** Hongbo Chen, Eldan Cohen, Dulaney Wilson, Myrtede Alfred

**Affiliations:** 1 Department of Mechanical & Industrial Engineering Faculty of Applied Science & Engineering University of Toronto Toronto, ON Canada; 2 Department of Public Health Sciences College of Medicine Medical University of South Carolina Charleston, SC United States

**Keywords:** accident, accidents, black box, classification, classifier, collaboration, design, document, documentation, documents, explainability, explainable, human-AI collaboration, human-AI, human-computer, human-machine, incident reporting, interface design, interface, interpretable, LIME, machine learning, patient safety, predict, prediction, predictions, predictive, report, reporting, safety, text, texts, textual, artificial intelligence

## Abstract

**Background:**

Adverse events refer to incidents with potential or actual harm to patients in hospitals. These events are typically documented through patient safety event (PSE) reports, which consist of detailed narratives providing contextual information on the occurrences. Accurate classification of PSE reports is crucial for patient safety monitoring. However, this process faces challenges due to inconsistencies in classifications and the sheer volume of reports. Recent advancements in text representation, particularly contextual text representation derived from transformer-based language models, offer a promising solution for more precise PSE report classification. Integrating the machine learning (ML) classifier necessitates a balance between human expertise and artificial intelligence (AI). Central to this integration is the concept of explainability, which is crucial for building trust and ensuring effective human-AI collaboration.

**Objective:**

This study aims to investigate the efficacy of ML classifiers trained using contextual text representation in automatically classifying PSE reports. Furthermore, the study presents an interface that integrates the ML classifier with the explainability technique to facilitate human-AI collaboration for PSE report classification.

**Methods:**

This study used a data set of 861 PSE reports from a large academic hospital’s maternity units in the Southeastern United States. Various ML classifiers were trained with both static and contextual text representations of PSE reports. The trained ML classifiers were evaluated with multiclass classification metrics and the confusion matrix. The local interpretable model-agnostic explanations (LIME) technique was used to provide the rationale for the ML classifier’s predictions. An interface that integrates the ML classifier with the LIME technique was designed for incident reporting systems.

**Results:**

The top-performing classifier using contextual representation was able to obtain an accuracy of 75.4% (95/126) compared to an accuracy of 66.7% (84/126) by the top-performing classifier trained using static text representation. A PSE reporting interface has been designed to facilitate human-AI collaboration in PSE report classification. In this design, the ML classifier recommends the top 2 most probable event types, along with the explanations for the prediction, enabling PSE reporters and patient safety analysts to choose the most suitable one. The LIME technique showed that the classifier occasionally relies on arbitrary words for classification, emphasizing the necessity of human oversight.

**Conclusions:**

This study demonstrates that training ML classifiers with contextual text representations can significantly enhance the accuracy of PSE report classification. The interface designed in this study lays the foundation for human-AI collaboration in the classification of PSE reports. The insights gained from this research enhance the decision-making process in PSE report classification, enabling hospitals to more efficiently identify potential risks and hazards and enabling patient safety analysts to take timely actions to prevent patient harm.

## Introduction

Since the publication of the seminal report on patient safety—*To Err Is Human* [1], the importance of preventing adverse events in health care has been widely recognized. Adverse events refer to unintended or unexpected incidents that occur during hospital care that cause harm to a patient [2]. Common adverse events include complications, falls, and medication errors. These events can lead to prolonged hospital stays, permanent harm to patients, life-saving interventions, or even contributing to patient deaths [2,3]. Unfortunately, adverse events remain one of the top 10 leading causes of death and disability worldwide, resulting in 251,454 deaths annually in the United States alone [4]. In Organization for Economic Cooperation and Development (OECD) countries, 15% of total hospital activity is the direct result of adverse events [5]. The global cost of adverse events has been estimated at 42 billion USD annually [6].

Patient safety event (PSE) reporting systems, also called incident reporting systems, have been widely adopted in hospitals across the world as part of their efforts to mitigate adverse events and improve patient safety [7,8]. Multiple nations, including Canada, Japan, England, and Norway, have made it mandatory for hospitals to establish and maintain a PSE reporting system, either with individual health care systems or through centralized national incident reporting platforms [9]. The primary purpose of the PSE reporting system is to provide health care organizations with a centralized system for tracking and analyzing PSEs, thereby facilitating continuous learning and maintaining a record of PSEs for risk assessment and prevention [7,10]. PSE reporting systems are tools that allow frontline health care personnel to voluntarily report adverse events, near-misses, and unsafe conditions [11]. Each PSE report includes structured data, such as event types, patient harm level, date, and location of the event, as well as unstructured data, including a free-text section that contains the factual description of the event and the patient’s outcome [12]. Following submission, PSE reports are reviewed by relevant hospital staff, such as risk managers, patient safety analysts, nurse managers, physicians, and biomedical engineers, to identify areas for patient safety and quality improvement within the hospital [13].

Accurately classifying PSE reports into their appropriate event type is crucial to ensure that these reports are directed to the relevant patient safety analyst, support organizational learning, identify patterns and trends in adverse events, and ultimately prioritize measures to reduce adverse events [14,15]. An event type refers to a specific class of events that share common characteristics [16]. Examples of event types include falls, medication-related issues, and diagnosis errors [17,18]. PSE reporting systems may have upwards of 20 categories of events. The formulation of these classification taxonomies generally involves systematically grouping PSE reports based on common characteristics [19]. The descriptions of event types are not always readily accessible to PSE reporters and patient safety analysts [15]. Previous studies have found that the classification of PSE reports is inconsistent depending on the reporter’s profession, interpretation of the adverse event, and understanding of the PSE classification taxonomy [15,20]. Furthermore, 25% of PSE reports are labeled with vague or nonspecific categories such as “miscellaneous” and “other” and require time-consuming retrospective analysis for reclassification [21]. These problems are further exacerbated by the growing volume of PSEs reported [18,22]. For instance, hospitals in the state of New South Wales in Australia reported close to 195,000 PSEs in 2020 [23], while there were approximately 2.3 million PSEs reported to the National Reporting and Learning System in England from April 2021 to March 2022 [24].

In light of these challenges, it is imperative to find an efficient solution to ensure the reliable classification of PSE reports. Recent studies have used static text representations and supervised machine learning (ML) techniques to automate the PSE report classification [17,25,26]. However, static text representations ignore the ordering of the words and do not account for the differences in word meaning across different contexts. These limitations may result in suboptimal classification performance. With the emergence of deep learning, contextual text representation produced from transformer-based deep learning models has achieved state-of-the-art performance on a wide range of natural language processing tasks, including text classification [27]. The contextual representation of each word is based on its surrounding context within the text, allowing for a more accurate understanding of its usage across different contexts and facilitating knowledge transfer across languages [28]. Therefore, using contextual text representation in training ML classifiers presents a promising opportunity for achieving a more precise classification of PSE reports.

The integration of ML models into PSE reporting systems has important implications for human–artificial intelligence (AI) collaboration, given the roles of the incident reporter (front end) and patient safety analyst (backend). Various approaches for using ML classifiers can be developed, including at different levels of automation; however, unifying the strengths of both human expertise and AI offers the most promising route for effective implementation [29-31]. A crucial determinant for successfully implementing the human-AI collaboration approach is decision transparency [32,33], which is often referred to as explainability. Explainability is the concept that an ML model’s prediction can be explained in a way that human operators can comprehend and reconstruct the model’s reasoning [33]. Incorporating explainability techniques in human-AI collaboration is paramount as it facilitates a deeper understanding of the factors influencing the predictions, thereby fostering trust and understanding between human experts and AI systems. Therefore, embedding explainability into the human-AI collaboration holds significant potential for enhancing PSE report classification.

The main aim of this study is to examine the efficacy of contextual text representation in improving the accuracy of PSE report classification. To accomplish this, we trained, evaluated, and compared various ML classifiers with both static and contextual text representations. Additionally, we developed an interface to illustrate the integration of the ML classifier in an event reporting system to support human-AI collaboration for PSE report classification. Moreover, we enhanced the explainability of the ML classifiers by using an explainable AI technique. Furthermore, we have investigated the ML classifier’s performance under 2 conditions, differentiated by whether the explanation is valid for the predicted event type. Based on this analysis, we offer recommendations for optimizing human-AI collaboration in the context of PSE report classification.

## Methods

### Data Collection

The data set for this study was obtained from a large academic hospital located in the Southeastern United States. A total of 861 PSE reports from the labor and delivery and mother-baby units were extracted from the PSE reporting system from January 1, 2019, to December 31, 2020. Each PSE report was assigned to a single event type from a set of 25 classes, such as complication of the surgery, fall, medication-related, and supply issues. The ML classifiers were trained exclusively on PSE reports from the 7 most frequently occurring event types. This selection was intended to create a more balanced training data set to reduce sampling bias and the risk of overfitting. The selected PSE reports used for training ML classifiers constitute approximately 72.8% (627/861) of the extracted reports (Table 1).

**Table 1:** Prevalence of patient safety event reports by event type in this study.

Event type	Extracted reports (n=861), n (%)
Care coordination or communication	186 (21.6)
Laboratory test	122 (14.2)
Medication related	89 (10.3)
Omission or errors in assessment, diagnosis, and monitoring	67 (7.8)
Maternal	58 (6.7)
Equipment or devices	56 (6.5)
Supplies	49 (5.7)
Total	627 (72.8)

### Data Preprocessing

The free-text section of PSE reports was preprocessed before feeding into ML classifiers as input features. The preprocessing procedures include text normalization, feature extraction, data splitting, and data augmentation (Multimedia Appendix 1 [28,34-39]).

### Classifier Training

A range of ML classifiers, including multinomial logistic regression (MLR), support vector machine (SVM), extreme gradient boosting, light gradient boosting, random forest (RF), *k*-nearest neighbor (KNN), and multilayer perceptron, were used for the classification of PSE reports. While SVM is a binary classifier, it is also capable of performing multiclass classification using the one-versus-one strategy. This involves treating the multiclass classification problem as a series of binary classification problems, creating *n × (n – 1) / 2* binary classifiers for each pair of classes, where *n* represents the total number of classes, and the final classification is based on the majority vote of all binary classifiers. Extreme gradient boosting, light gradient boosting, and RF are tree-based ensemble algorithms that are commonly used in text classification tasks [17,40]. The KNN classifier predicts the class of a data point based on the majority class among its nearest neighbors in the training data set. Multilayer perceptron is a feedforward neural network consisting of multiple layers of interconnected neutrons and trained using backpropagation.

To optimize the performance of ML classifiers, we used the 5-fold cross-validation grid search technique to identify the best combination of hyperparameters. During this process, a range of values of important hyperparameters (ie, regularization strength) is assessed with 5-fold cross-validation. For each combination of hyperparameters, the training set is randomly split into 5 distinct folds, and then the ML classifier is trained and evaluated 5 times, picking a different fold for evaluation every time and training on the remaining 4 folds. The optimized combination of hyperparameters is determined based on the average performance of the classifier on the *F*_1_-score across the 5-fold cross-validation runs.

### Classifier Evaluation

We evaluated the performance of the trained classifiers on the testing set with standard classification metrics, including accuracy, precision, recall, *F*_1_-score, and area under the receiver operating characteristic curve. We also evaluated classifiers on top-2 accuracy, which measures the proportion of predictions where the correct event type is among the top 2 highest probability event types predicted by the classifier. The definitions and mathematical formulas of the evaluation metrics are shown in Multimedia Appendix 2. Each of these metrics provides a distinct perspective on the performance of the classifier, and collectively, they offer a comprehensive understanding of how well the classifier is functioning. Since we framed PSE report classification as a multiclass text classification problem, the precision, recall, *F*_1_-score, and area under the receiver operating characteristic curve are computed for each class and combined using a weighted average where the weights correspond to the number of data points in each class.

### Development and Assessment of Explainability

As the contextual text representation is generated from transformer-based neural network, which has a black box nature, we used the local interpretable model-agnostic explanations (LIME) technique to analyze the top-performing ML classifier trained with the contextual text representation. LIME is a post hoc, local perturbation technique that provides the explanation for a single prediction. LIME generates perturbed data by randomly removing words from a text document and trains a locally explainable model with perturbed data to simulate the original classifier’s prediction [41]. By measuring how the classifier’s prediction changes under these perturbations, LIME reflects the contributions of each word to the prediction. The importance of each word can then be assessed for a single prediction, revealing whether the ML classifier has learned to use relevant words for classifying PSE reports. We used LIME to generate explanations for the top-performing classifier’s prediction, specifically by highlighting the words that the classifier deems influential for the prediction. We presented 3 distinct cases: one where the classifier effectively leveraged relevant words for accurate prediction, another where it failed to do so, and a final case that illustrated the explanation for a misclassification. In addition, we analyzed the top 5 most prevalent words identified by LIME for each event type.

A total of 2 human factors graduate students were recruited to assess the quality of the LIME explanations. For each PSE report in the test data set, the reviewers were asked to determine independently if any of the highlighted words were relevant to the predicted event type. Based on these evaluations, the reports were then categorized into 2 distinct groups: those in which the highlighted terms were deemed relevant to the predicted event types and those where they were deemed irrelevant. Discrepancies were resolved through discussions. The interrater reliability index (Cohen κ) was calculated to quantify the level of agreement between the reviewers. The ML classifier’s accuracy and *F*_1_-score were evaluated for these 2 groups of PSE reports. A subsequent comparison will explore the influence of explanation quality on prediction reliability.

### Interface Development

In the typical workflow of PSE report classification, reporters need to provide a narrative description of the event as well as key attributes such as the event type, level of harm, date, and location of the event. Subsequent to this initial classification, the patient safety analyst will review the submitted report and decide if it needs to be recategorized to better reflect the nature of the event [17,42]. To support efficient and reliable categorization, the classifier will need to provide reporters with real-time support during the reporting process. We developed a PSE reporting interface to illustrate the integration of the ML classifier and the LIME explainability technique. In the design, the ML classifier provides multiple high-probability event types along with explanations for its prediction and allows the user to select the most appropriate event type. The interface was developed in Figma [43] and designed using guidance from previous research on incident reporting systems, including question type, mandatory and optional questions, and taxonomy for event type and harm level [44,45].

### Ethical Considerations

The study was approved by the Medical University of South Carolina Hospital’s institutional review board (Pro00105892). Following data extraction, PSE reports were anonymized in accordance with privacy regulation guidelines.

## Results

### Performance Comparison

We evaluated the trained ML classifier’s classification performance on both static and contextual text representations (Multimedia Appendix 3). The performance of the top-performing ML classifier trained with static and contextual text representations is shown in Table 2. Our results showed that for static text representation, the MLR classifier trained with term frequency–inverse document frequency (TF-IDF) achieved the best performance, with an *F*_1_-score of 0.631 and an accuracy of 66.7% (84/126). On the other hand, for contextual text representation, the SVM classifier trained with RoBERTa-base outperformed others, with an *F*_1_-score of 0.753 and an accuracy of 75.4% (95/126). The SVM classifier trained with RoBERTa-base showed a 19.3% elative improvement in *F*_1_-score and a 13% (11/85) relative improvement in accuracy compared to the MLR classifier trained with TF-IDF for contextual text representation. In addition, we compared the accuracy (95/126, 75.4%) and top 2 accuracy (107/126, 84.9%) of the SVM classifier trained with RoBERTa-base and observed that 9.5% (12/126) of PSE reports’ true event type was predicted as the second highest probability event type by the classifier, which represents 39% (12/31) of misclassified PSE reports.

**Table 2:** Performance of top-performing ML classifiers trained with static and contextual text representations.

Metric	Top-performing ML^a^ model trained with the static text representation				Top-performing ML model trained with the contextual text representation		
	Performance	ML classifier	Text Representation	Performance		ML classifier	Text Representation
Accuracy (%)	66.67	MLR^b^	TF-IDF^c^	75.40		SVM^d^	RoBERTa-base
Top 2 accuracy (%)	85.71	MLR	TF-IDF	88.10		MLP^e^	xlm-RoBERTa-base
Precision	0.707	KNN^f^	TF-IDF	0.757		SVM	RoBERTa-base
Recall	0.667	MLR	TF-IDF	0.754		SVM	RoBERTa-base
*F*_1_-score	0.631	MLR	TF-IDF	0.753		SVM	RoBERTa-base

^a^ML: machine learning.

^b^MLR: multinomial logistic regression.

^c^TF-IDF: term frequency–inverse document frequency.

^d^SVM: support vector machine.

^e^MLP: multilayer perceptron.

^f^KNN: *k*-nearest neighbor.

### Performance on Classifying Individual Event Types

We analyzed the performance of the SVM classifier trained with RoBERTa-base on individual event types (Table 3). The *F*_1_-score measure for different event types ranged from 0.958 (laboratory test) to 0.400 (omission or errors in assessment, diagnosis, and monitoring).

**Table 3:** Performance of support vector machine+RoBERTa-base on the individual event type.

Event type	Precision	Recall	*F*_1_-score
Care coordination or communication	0.721	0.838	0.775
Laboratory test	1.000	0.920	0.958
Medication related	0.765	0.722	0.743
Omission or errors in assessment, diagnosis, and monitoring	0.417	0.385	0.400
Maternal	0.750	0.750	0.750
Equipment or devices	0.700	0.636	0.667
Supplies	0.778	0.700	0.737

**Figure 1:**
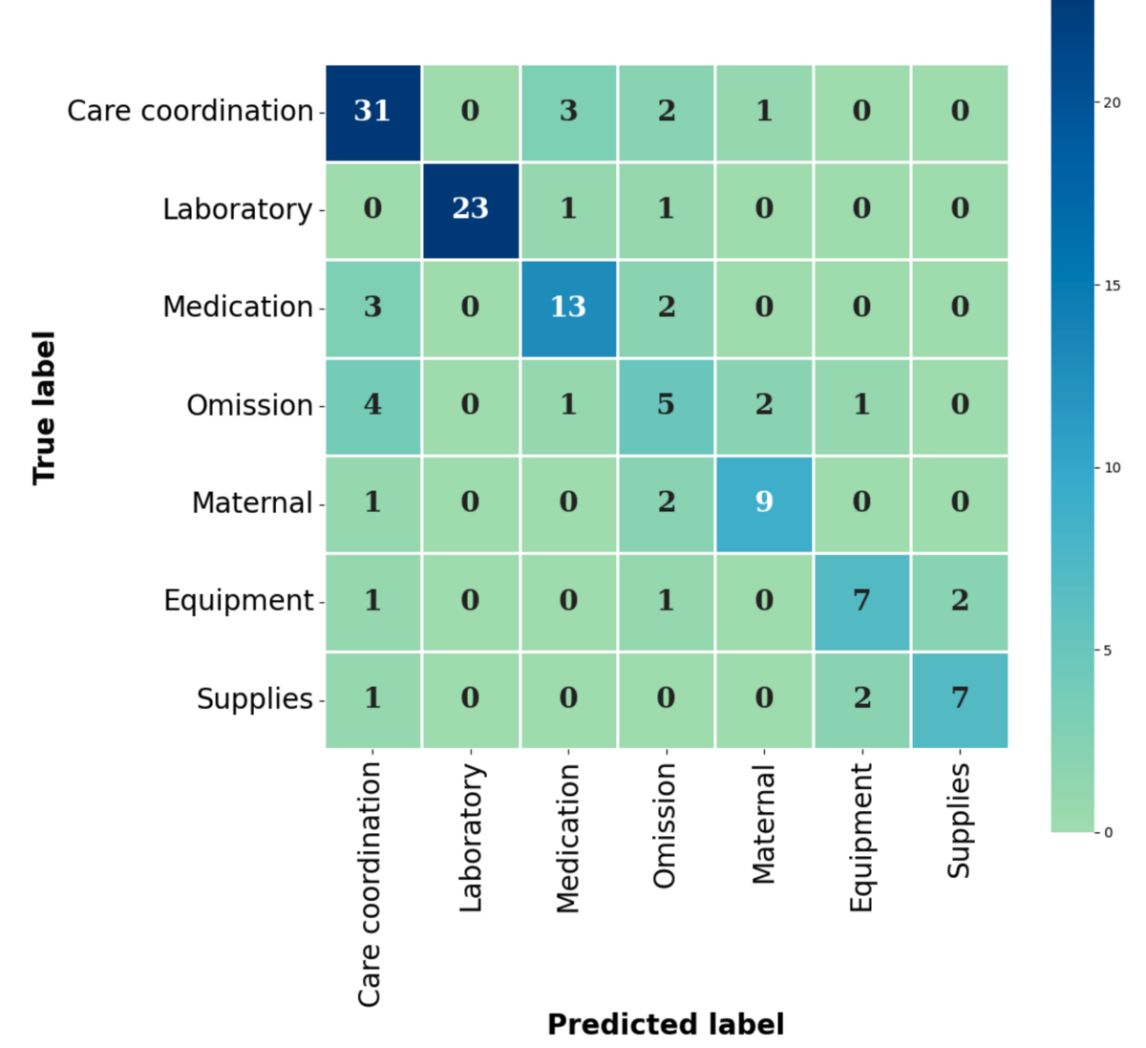
Confusion matrix for the testing set evaluation with a support vector machine classifier trained with RoBERTa-base.

Figure 1 shows the confusion matrix for the SVM classifier trained with RoBERTa-base evaluated on the test set. A confusion matrix is a table that visualizes the performance of a classifier. The main diagonal value is the number of PSE reports that have been classified as true event types, whereas off-diagonal values are the number of PSE reports that have been wrongly classified. While the classifier was able to classify the majority of event types of PSE reports correctly, there is a consistent misclassification of the omission or errors in assessment, diagnosis, or monitoring PSE report as the care coordination or communication (coordination) event type.

### LIME-Based Explainability Analysis

**Figure 2:**
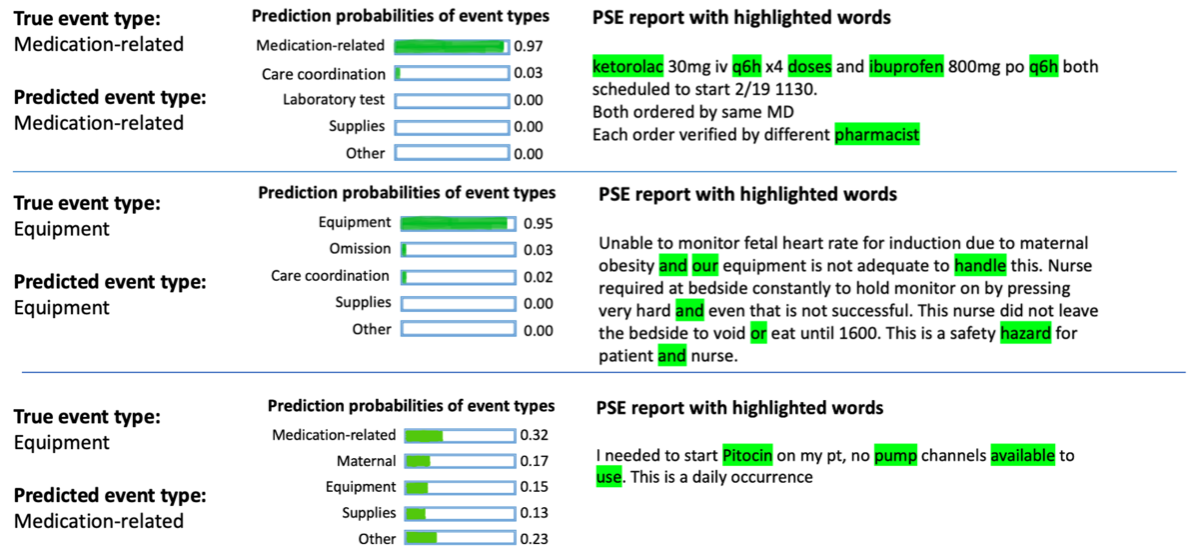
Local interpretable model-agnostic explanations of support vector machine classifiers trained with RoBERTa-base. MD: medical doctor; PSE: patient safety event; pt: patient.

We used LIME to evaluate whether the SVM classifier trained with RoBERTa-base has leveraged informative words for classification. Figure 2 presents 3 examples of explanations for the classifier’s predictions. At the top of Figure 2, LIME identified “ketorolac,” “ibuprofen,” and “doses” from the PSE report as important words for classifying the report into the medication-related event type, which is reasonable given the report’s association with incorrect medication doses. Conversely, in the middle of Figure 2, LIME highlighted “our,” “handle,” and “or” from the text as important words for classifying the report into the equipment or device event type. Although the predicted event type was correct, the classifier relied on irrelevant words for the classification. At the bottom of Figure 2, a case of misclassification is shown. LIME highlighted “pitocin,” “pump,” “available,” and “use” as influential words for classifying the PSE report into medication-related event type when it belongs to the equipment class. In addition, for each event type, we extract the 5 most prevalent words that were deemed important for the classifier’s prediction across the whole data set (Table 4). This inclusion of stop words (ie, “was,” “not,” and “till”) among influential terms, as shown in Table 4, demonstrated that the classifier does not always rely on relevant words for making classifications.

**Table 4:** The 5 most prevalent and important words for each event type were derived from the support vector machine classifier trained with RoBERTa-base.

Event type	Prevalent influential words highlighted by local interpretable model-agnostic explanations
Care coordination or communication	requested, delayed, patient, not, follow
Laboratory test	specimen, lab, labels, collection, results
Medication related	patches, doses, orders, medication, pitocin
Omission or errors in assessment, diagnosis, and monitoring	warning, patient, was, till, late
Maternal	baby, hysterectomy, stable, pumping, hemorrhage
Equipment or devices	instruments, trays, notified, malfunctioning, faulty
Supplies	vendor, sterile, available, needed, OR

After reviewing the LIME explanations for each PSE report in the test data set, 73.8% (93/126) of the reports were categorized into a subset where at least 1 highlighted word was deemed relevant to the predicted event type. The remaining reports comprised a second subset where no highlighted words were relevant. The interrater reliability index measured by Cohen κ between the 2 reviewers was 0.83, indicating substantial agreement. Table 5 presents the performance of the top-performing ML classifier for both subsets. For the first subset, the classifier achieved an accuracy of 84% (78/93) and an *F*_1_-score of 0.825. In contrast, the second subset showed a classifier accuracy of 52% (17/33) and an *F*_1_-score of 0.549.

**Table 5:** Performance of a top-performing machine learning classifier on reports that have relevant words highlighted and reports with irrelevant words highlighted.

Metric	PSE^a^ reports with relevant words highlighted	PSE reports with irrelevant words highlighted
Number of PSE reports, n	93	33
Percentage of test data set (%)	73.81	26.19
Accuracy (%)	83.87	51.51
*F*_1_-score	0.825	0.549

^a^PSE: patient safety event.

### PSE Reporting System Interface

**Figure 3:**
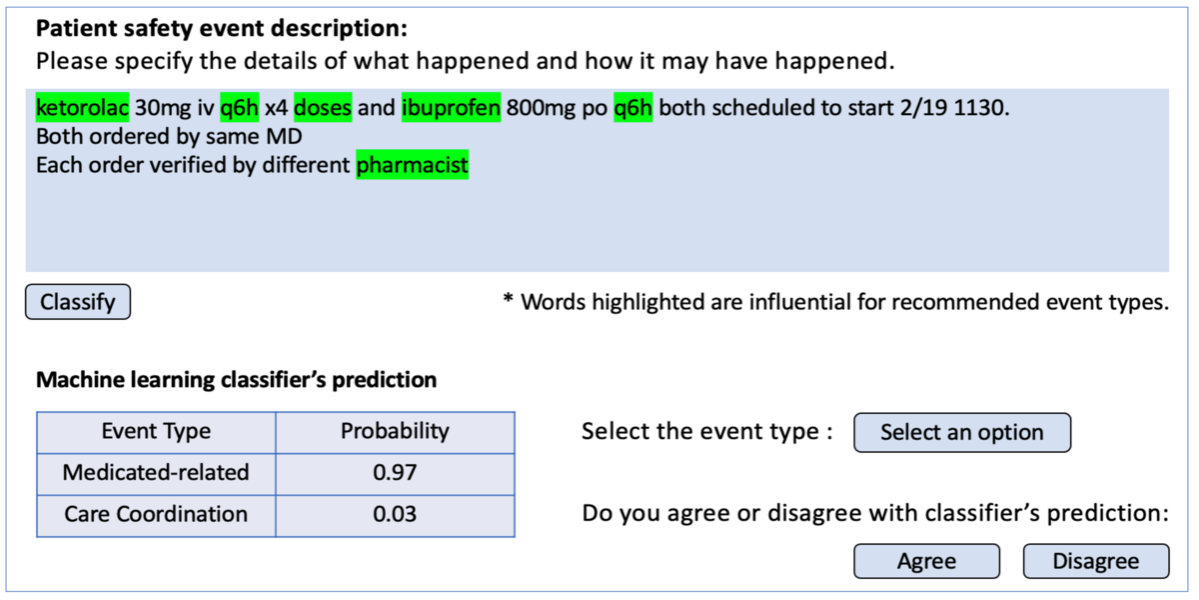
Interface visualization of a patient safety event report classifier coupled with the local interpretable model-agnostic explanations technique. MD: medical doctor.

We designed an event reporting interface that integrates both the ML classifier and the LIME explainability technique. Figure 3 shows the event classification screen, where reporters enter a narrative description of the event after providing the details of the event, including date, time, unit, and information about the patient and reporter. Before describing the event in narrative form, reporters also choose among factors that contributed to the incident and the level of harm experienced by the patient. Once the reporter enters their narrative and selects the “classify” button, the system activates the ML classifier. Subsequently, the interface displays the top 2 most probable event types, along with their associated probability distributions, in the lower left section. Simultaneously, the LIME technique will identify influential words that significantly contributed to the predicted event type, highlighting these words in green in the upper section of the dashboard. Based on the predicted event types and words highlighted for their influence on the prediction, the reporter may select the most suitable event type from a drop-down menu located in the lower-right section of the dashboard. Following this selection, reporters are queried on whether they agree with the classifier’s prediction, and the collected data can be used to guide subsequent refinement of the ML classifier.

## Discussion

### Overview

PSE event reporting systems are commonly used in health systems and hospitals across the world [46]. Data collected in PSE reporting systems drive quality improvement and patient safety efforts and supports regulatory reporting requirements for hospitals. The erroneous classification of PSE reports can impede the learning capabilities of the PSE reporting system, leading to suboptimal performance in detecting and preventing potential patient safety hazards [20]. It can also result in a substantial time cost for reclassifying PSE reports and compromise the integrity of a PSE database when analysts are investigating trends in events to develop effective solutions [17]. Previous studies have trained ML classifiers with static text representations for automatic PSE classification [12,17,25,26]. This study aimed to investigate whether using contextual text representations can further improve the accuracy of classifying PSE reports. We trained and evaluated a range of ML classifiers using both static and contextual text representations. To the best of our knowledge, this is the first time that contextual text representation has been used for training ML classifiers for PSE report classification. We analyzed the confusion matrix of the top-performing classifier to identify prevalent misclassified event types. Furthermore, aiming for more accurate and reliable PSE report classification, we incorporated an explainability technique to support human-AI collaboration and designed an interface to illustrate the possible integration of the ML classifier in PSE reporting systems.

### Principal Findings

In this study, we extensively investigated the potential of using contextual representation for improving PSE report classification. The leading classifier trained with the static text representation (MLR trained with TF-IDF) was able to achieve an accuracy of 66.7% (84/126). This accuracy considerably exceeds the baseline accuracy of 29.4% (37/126), which involves classifying all PSE reports into the majority event type. However, using contextual text representation proved more efficacious. The SVM trained with contextual text representation (RoBERTa-base) was able to achieve an accuracy of 75.4% (95/126), reflecting a 13% (11/84) relative improvement in accuracy compared to the best-performing classifier trained with static text representation. While the achieved accuracy of 75.4% may not appear outstanding in isolation, it represents a significant advance compared with static text representations and exceeds the baseline, given the limited size of the data set. The improvement in classifier performance can be attributed to the use of contextual text representations, which can capture not only the meaning of individual words but also the complex and subtle ways in which words interact with each other in a specific context. Therefore, contextual text representation overcomes some limitations of static text representation, which relies primarily on word frequency and co-occurrence to represent text. Moreover, contextual text representation does not require explicit text normalization while also avoiding issues associated with high-dimensionality and sparsity commonly found in static text representations. Hence, when training ML classifiers for PSE reporting systems, contextual text representation should be prioritized over static text representation to ensure the highest level of accuracy in classifying PSE reports.

As part of our investigation, we evaluated the performance of the top-performing classifier trained with contextual text representation on individual event types. While the classifier demonstrated impressive performance in accurately classifying laboratory test PSE reports (*F*_1_-score=0.958), it struggled with classifying omissions or errors in assessment, diagnosis, and monitoring PSE reports, resulting in an unsatisfactory *F*_1_-score of 0.400. To investigate this discrepancy, we analyzed the confusion matrix for the classifier and discovered that omissions or errors in assessment, diagnosis, and monitoring PSE reports were frequently misclassified as the coordination event type. This misclassification can be attributed to the multiclass nature of PSE reports. For example, a failure to document the removal of a patient’s epidural catheter (omission or errors in assessment, diagnosis, and monitoring) could lead to a medication ordered by a physician (such as Lovenox) being withheld by the pharmacy due to a complication risk (coordination). On the other hand, the laboratory test is a more distinct event type in comparison to the other event types, and the classifier was able to correctly classify the majority of these reports. The observation obtained from the confusion matrix implies that PSE reports can potentially have more than 1 event type. This finding is consistent with previous studies [25,26]. The finding also underscores the need for further refinement in the development of the PSE taxonomy to create more distinctive event types. Another potential solution for addressing the multiclass nature of PSEs is to enable multiple event-type assignments [47]. Alternatively, the ML classifier can provide several probable event types, allowing the user to select the most appropriate one. We evaluated the top 2 accuracy of the top-performing ML classifier trained with contextual text representation and observed that 39% (12/31) of misclassified PSE reports’ true event type was predicted as the second-highest probability event type by the classifier. The finding suggests that there is a greater chance for the ML classifier to provide the correct event type when considering multiple options. As event reporting systems usually encompass over 20 event types, which can be difficult to memorize or access [17], narrowing down the PSE report’s potential event types to a smaller range also reduces the cognitive workload for PSE reporters during the classification process [48] and enhances the efficiency of reclassifying PSE reports for patient safety analysts.

We used LIME to showcase 3 predictions’ explanations and demonstrated cases where the ML classifier used informative words for classifying the PSE report and where it used irrelevant words for classification. These results highlight the importance of not solely relying on the ML classifier’s prediction and underscore the need for explainability and transparency in using the ML classifier for PSE report classification. Additionally, we showed the top 5 most prevalent words the ML classifier deemed important in the PSE reports for each event type. These words are indicative of the prevalent themes and issues within specific event types. Understanding the context and relationships between these prevalent informative words and specific event types can potentially provide valuable insights into the factors contributing to different types of PSEs. Furthermore, we have evaluated the top-performing ML classifier’s performance on 2 subsets of PSE reports, differentiated by whether the highlighted word by LIME is relevant to the predicted event type. Our findings reveal that the majority of PSE reports (93/126) have at least 1 relevant word highlighted, with the classifier achieving an accuracy of 84% (78/93) on these reports. Conversely, accuracy drops to 52% (17/33) when irrelevant words are highlighted. Such a disparity in performance emphasizes the necessity for additional scrutiny from reporters and patient safety analysts, particularly when dealing with PSE reports that have irrelevant words highlighted.

While previous research has focused on the development of ML classifiers, none of these previous works have investigated the potential integration of the classifier within the PSE reporting system in a manner that aligns with the workflow of the front-end reporter. We designed an interface to demonstrate the feasibility of a collaborative human-AI approach for event categorization. The interface provides the PSE reporter with multiple probable event types and associated explanations for the ML classifier’s prediction. This approach aligns with the principles of level 2 automation, where ML classifiers aids human decision-making rather than fully automating it [49]. This collaboration optimally combines human expertise with ML capabilities, potentially reducing cognitive workload and memorization of the taxonomy while also reducing the risks associated with overreliance on automation. Numerous studies have shown that the human-AI collaboration approach can improve the decision-making process [50-52], indicating its potential for enhancing PSE report classification. Furthermore, the interface also integrates the LIME explainability technique, which offers real-time insights into the rationale for the probable event types. Given the role of reporters and patient safety analysts in the incident reporting process, the use of explainability techniques can also increase trust in the recommendation provided by the ML classifier as it provides transparent and interpretable reasoning for the classification decisions [50,51]. Using LIME to highlight top informative words in real time for a PSE report can assist PSE reporters by emphasizing keywords in their narratives that are linked to the proposed classification. Highlighting informative words can also facilitate patient safety analysts working at the back end by providing insights into why a specific event type was chosen for classification. Such transparency not only clarifies current recommendations but also guides analysts in identifying influential terms for future report classifications. Previous research has illustrated the value of automation transparency in supporting appropriate levels of trust in the system, including decision support systems [32]. Additionally, regularly checking the explanations of the ML classifier’s prediction enables continuous monitoring of the classifier’s performance, identification of issues, and refinement [52]. As we have only designed the interface, additional research is needed to test the effectiveness of this approach in PSE report classification. Assessing the interface’s impact on cognitive workload and decision-making accuracy is essential for ensuring its usability and adoption in the event reporting system. We plan to undertake a usability testing study with health care professionals in a subsequent study.

### Comparison With Previous Work

Research into the use of ML classifiers for the automation of PSE report classification has been relatively scarce. Wang et al [26] used logistic regression and SVM with the binary count, term frequency, and TF-IDF text representation to classify ten types of PSE reports, reaching an *F*_1_-score as high as 0.783. However, they used a considerably larger data set (n=2860). Fong et al [17] achieved an accuracy rate of 92.0% (284/309) when they examined the usage of an ML classifier for classifying miscellaneous PSE reports using SVM, RF, and logistic regression with TF-IDF [17]. They also used a much larger data set (n=70,051). Ong et al [12] investigated the feasibility of using an ML classifier to automatically classify 2 types of PSE reports, including inadequate clinical handover and incorrect patient identification. They used Bag of Words model for text representation and trained both SVM and naive Bayes on classifying PSE reports, reaching accuracy as high as 98% (364/372). However, they framed the problem as a binary classification problem, which inherently has a higher baseline accuracy compared to our investigation. In this study, we’ve performed an in-depth comparative analysis with the available PSE data set and compared the established methods of classifying PSE reports and our novel method of using contextual text representations for classification. Our findings reveal that our proposed method outperforms the traditional models in terms of accuracy (ie, 84/126, 66.7% vs 95/126, 75.4%) and *F*_1_-score (ie, 0.631 vs 0.753). This underlines the significance of our approach and its potential to advance the field of using ML classifiers for PSE report classification.

### Limitations

There are several limitations to this study. First, the PSE reports used to train the ML classifiers were obtained from the maternal care units of a single hospital in the United States; therefore, the classifier might not generalize well to other settings. Second, this research’s scope was constrained by the limited amount of PSE report data, and only 7 prevalent classes were incorporated for training the ML classifiers. The restricted quantity of PSE reports might also result in an underestimation of the ML classifier’s actual capabilities [12]. Third, the quality of the LIME explanations was assessed by 2 graduate students; thus, further investigation is needed for a more robust validation of explanation quality. Furthermore, we have not yet empirically tested the interface for potential decision-making biases it may introduce.

Future research should investigate the performance of ML classifiers trained with contextual text representations on a larger and more diverse data set. Additionally, while we plan to refine the interface and test whether it supports event classification, future research can continue to investigate the appropriate way of incorporating the ML classifier into the reporting and reviewing workflow of PSE report classification and examine various human-AI collaboration approaches. Future studies should explore the potential biases (ie, automation bias) that the interface may introduce into the analysts’ decision-making process.

### Conclusions

Improving the precision of PSE report classifications is a multifaceted task, involving both the refinement of the event type taxonomy and adequate training of hospital staff on the event reporting system. Despite these challenges, ML classifiers offer substantial potential to support accurate classification throughout the reporting and reviewing process. The findings of this study contribute to the advancement of ML classifiers for PSE report classification by demonstrating the superior performance of contextual text representation over static text representations in achieving more accurate classification outcomes. The integration of explainability techniques in ML classifiers fosters trust in their usage and provides valuable insights for informed decision-making and potential adjustments to the classifier. An event reporting interface that integrates an ML classifier with collaborative decision-making capabilities offers the potential to achieve an efficient and reliable PSE report classification process. These approaches can ultimately help hospitals identify risks and hazards promptly and take timely and informed actions to mitigate adverse events and reduce patient harm.

## Appendix

Multimedia Appendix 1 Data preprocessing procedures.

Multimedia Appendix 2 Evaluation metrics for examining patient safety event machine learning classifiers.

Multimedia Appendix 3 The performance of machine learning classifiers in classifying patient safety event report event type.

## Abbreviations

AI: artificial intelligence
KNN: k-nearest neighbor
LIME: local interpretable model-agnostic explanations
ML: machine learning
MLR: multinomial logistic regression
OECD: Organization for Economic Cooperation and Development
PSE: patient safety event
RF: random forest
SVM: support vector machine
TF-IDF: term frequency–inverse document frequency
