# Supplementary material for: A Machine Learning Approach with Human-AI Collaboration for Automated Classification of Patient Safety Event Reports: Algorithm Development and Validation Study
Source: JMIR Hum Factors. 2024 Jan 25;11:e53378. doi: 10.2196/53378 (PMC10853856; doi:10.2196/53378)
Supplement: Multimedia Appendix 1 [file humanfactors_v11i1e53378_app1.docx]

## Multimedia Appendix – 1

Data preprocessing procedures

#### Text normalization

Two types of features were extracted from the free text section of PSE reports including static text representation and contextual text representation. Static text representation requires text normalization to recognize the same word in different forms (i.e., singular vs. plural), this is achieved by a series of data cleaning procedures, including converting all words to lowercase, removing non-alphabetical characters, stop words, as well as applying stemming. In contrast, contextual text presentation does not require text normalization. This is because contextual text representation is generated by transformer-based NN that are trained on a substantial amount of raw text, making it resilient to variations in words such as casing, word tenses, and plurality [28,34].

#### Feature extraction

Post text normalization, three common static text representations were obtained including the bag of words (BOW), term frequency-inverse document frequency (TF-IDF), and global vectors (GloVe). BOW is a vector representation of the text document where the total number of occurrences of each word in the text document is used as a feature. TF-IDF is a vector format representation of a text document that reflects how relevant a word is to a text document in the entire dataset. The static text representation produced from BOW and TF-IDF in this study both used n-grams ranging from 1 to 3. GloVe represents each word with a vector that captures the semantic relationship between words [35] and the representation of the text document is then obtained by taking the average of each word’s vector.

Five contextual text representations were obtained by passing the free text section of PSE reports into pre-trained transformer-based NNs, including BERT-base, PubMed-BERT-base, xlm-RoBERTa-base, RoBERTa-large, and RoBERTa-base [36]. BERT is a transformer-based NN which stands for Bidirectional Encoder Representations from Transformers, it was trained in a self-supervision fashion using a large corpus of raw text with two tasks, masked language modeling and next sentence prediction [34]. BERT is capable of generating meaningful representations for text documents considering the semantic context within the text. RoBERTa is the optimized version of BERT, it builds on BERT and modifies key hyperparameters, removing the next sentence prediction task [37]. The difference between contextual text representations used in this study is the number of parameters in the transformer-based NN (i.e., RoBERTa-large: 255 million parameters vs. RoBERTa-base: 125 million parameters) and domain of raw text used to train the NN (i.e., PubMed-BERT-base is trained on abstracts from PubMed). All of the text representations have been standardized to ensure that the resulting ML classifier is not overly sensitive to the magnitudes of different features, which can help prevent issues such as overfitting.

#### Data splitting

To train the ML classifier for PSE reports classification, we randomly split the data into 80% for training and 20% for testing using stratified sampling, which is the standard methodology for data splitting when training ML classifiers. This approach helps to prevent overfitting and improve the generalizability of ML classifiers. By splitting the data in a stratified fashion, we ensured the testing set preserved the original distribution of event types, thus maintaining representative of the real-world scenario. Instead of creating a separate validation set, we used five-fold cross-validation during hyperparameter tuning to provide the ML classifier with more access to data during training.

#### Data augmentation

The distribution of PSE reports’ event type in this study is imbalanced in this study. Imbalanced data can lead to poor classification performance in the minority classes [38]. Therefore, we utilized the synthetic minority oversampling technique (SMOTE) to address the imbalance problem. SMOTE is an oversampling technique where synthetic data are created for the minority class to achieve a balanced distribution of classes, thus improving the classifier’s sensitivity to the minority class [39]. Only the training data set was augmented with SMOTE, while the testing set maintained its original distribution.
