# Supplementary material for: A Machine Learning Approach with Human-AI Collaboration for Automated Classification of Patient Safety Event Reports: Algorithm Development and Validation Study
Source: JMIR Hum Factors. 2024 Jan 25;11:e53378. doi: 10.2196/53378 (PMC10853856; doi:10.2196/53378)
Supplement: Multimedia Appendix 2 [file humanfactors_v11i1e53378_app2.docx]

## Multimedia Appendix - 2

### Evaluation metrics for examining PSE ML classifiers

#### Evaluation metrics definitions

- Accuracy - the overall percentage of PSE reports that the classifier has correctly classified.
- Top-2 accuracy - a variation of accuracy that measures the percentage of PSE reports where the true event type matches with one of the top two highest probability event types predicted by the classifier.
- Precision - answers how many PSE reports classified as one specific event type belong to that event type.
- Recall (also known as sensitivity) - represents the proportion of PSE reports that are correctly classified as their true event type.
- F1 - the harmonic mean of the precision and recall, which gives a whole picture of classifiers’ performance on both precision and recall.
- Area Under the Receiver Operating Characteristic Curve (AUCROC) - measures classifies’ ability to distinguish between event types by measuring the area under the curve which plots sensitivity versus (1-specificity).

#### Mathematical Definitions

True positive (TP): number of PSE reports correctly classified into a given event type.

False positive (FP): number of PSE reports falsely classified into a given event type.

True negative (TN): number of PSE reports correctly classified not to be in a given event type.

False negative (FN): number of PSE reports falsely classified as not being in a given event type.

N: Total number of PSE reports classified.

Accuracy = (TP+ TN)/N

Top-2 Accuracy = (Number of data has correct event types in the top 2 highest probability predicted event types) / N

Precision = TP/(TP+FP)

Recall = TP/(TP+FN)

F1 = 2*(Precision*Recall)/(Precision+Recall)
