# Supplementary material for: A Machine Learning Approach with Human-AI Collaboration for Automated Classification of Patient Safety Event Reports: Algorithm Development and Validation Study
Source: JMIR Hum Factors. 2024 Jan 25;11:e53378. doi: 10.2196/53378 (PMC10853856; doi:10.2196/53378)
Supplement: Multimedia Appendix 3 [file humanfactors_v11i1e53378_app3.docx]

## Multimedia Appendix-3

### The performance of ML Classifiers in Classifying PSE Report Event Type.

| Evaluation metrics | Text Representation / ML Classifier | MLR | SVM | XGB | RF | KNN | LGB | MLP |
| --- | --- | --- | --- | --- | --- | --- | --- | --- |
|  | | | | | | | | |
| Accuracy (%) | TF-IDF | *66.67* | 65.08 | 63.49 | 57.94 | 46.83 | 63.49 | 65.08 |
|  | BOW | 54.76 | 55.56 | 61.11 | 63.49 | 36.51 | 55.56 | 43.65 |
|  | GloVe | 53.17 | 62.70 | 53.97 | 58.73 | 56.35 | 54.76 | 60.32 |
|  | BERT-base | 62.70 | 60.32 | 51.59 | 52.38 | 42.86 | 55.56 | 65.87 |
|  | xlm-RoBERTa-base | 68.25 | 69.05 | 70.63 | 63.49 | 53.97 | 69.05 | 70.63 |
|  | RoBERTa-base | 66.67 | *75.40* | 66.67 | 65.87 | 50.00 | 69.05 | 68.25 |
|  | RoBERTa-large | 69.05 | 59.52 | 58.73 | 56.35 | 43.65 | 55.56 | 66.67 |
|  | PubMed-BERT-base | 55.56 | 65.87 | 59.52 | 61.11 | 51.59 | 61.11 | 62.70 |
|  | | | | | | | | |
| Top 2 Accuracy (%) | TF-IDF | *85.71* | 84.92 | 84.13 | 81.75 | 61.11 | 80.95 | 83.33 |
|  | BOW | 80.16 | 72.22 | 84.92 | 80.95 | 49.21 | 70.63 | 65.08 |
|  | GloVe | 74.60 | 79.37 | 73.02 | 79.37 | 65.87 | 76.98 | 82.54 |
|  | BERT-base | 83.33 | 78.57 | 73.02 | 71.43 | 55.56 | 78.57 | *85.71* |
|  | xlm-RoBERTa-base | 84.92 | 82.54 | 82.54 | 74.60 | 70.63 | 80.16 | *88.10* |
|  | RoBERTa-base | 84.13 | 84.92 | 81.75 | 80.95 | 65.08 | 80.16 | 84.92 |
|  | RoBERTa-large | 80.95 | 81.75 | 81.75 | 76.19 | 59.52 | 78.57 | 81.75 |
|  | PubMed-BERT-base | 82.54 | 85.71 | 76.98 | 77.78 | 62.70 | 83.33 | 83.33 |
|  | | | | | | | | |
| Precision | TF-IDF | 0.640 | 0.657 | 0.624 | 0.579 | *0.707* | 0.627 | 0.654 |
|  | BOW | 0.619 | 0.595 | 0.613 | 0.671 | 0.386 | 0.574 | 0.684 |
|  | GloVe | 0.540 | 0.629 | 0.571 | 0.600 | 0.627 | 0.569 | 0.621 |
|  | BERT-base | 0.641 | 0.635 | 0.526 | 0.524 | 0.565 | 0.552 | 0.658 |
|  | xlm-RoBERTa-base | 0.705 | 0.694 | 0.715 | 0.668 | 0.612 | 0.703 | 0.733 |
|  | RoBERTa-base | 0.706 | *0.757* | 0.666 | 0.665 | 0.642 | 0.701 | 0.710 |
|  | RoBERTa-large | 0.711 | 0.612 | 0.576 | 0.556 | 0.545 | 0.555 | 0.698 |
|  | PubMed-BERT-base | 0.571 | 0.661 | 0.616 | 0.648 | 0.652 | 0.627 | 0.648 |
|  | | | | | | | | |
| Recall | TF-IDF | *0.667* | 0.651 | 0.635 | 0.587 | 0.476 | 0.635 | 0.651 |
|  | BOW | 0.548 | 0.563 | 0.619 | 0.635 | 0.373 | 0.563 | 0.444 |
|  | GloVe | 0.532 | 0.627 | 0.540 | 0.595 | 0.571 | 0.548 | 0.611 |
|  | BERT-base | 0.627 | 0.611 | 0.516 | 0.524 | 0.429 | 0.563 | 0.659 |
|  | xlm-RoBERTa-base | 0.683 | 0.698 | 0.714 | 0.635 | 0.540 | 0.698 | 0.714 |
|  | RoBERTa-base | 0.667 | *0.754* | 0.667 | 0.659 | 0.500 | 0.698 | 0.690 |
|  | RoBERTa-large | 0.698 | 0.603 | 0.595 | 0.571 | 0.444 | 0.563 | 0.667 |
|  | PubMed-BERT-base | 0.556 | 0.659 | 0.603 | 0.619 | 0.516 | 0.619 | 0.627 |
|  | | | | | | | | |
| F1 | TF-IDF | *0.631* | 0.622 | 0.617 | 0.537 | 0.451 | 0.625 | 0.620 |
|  | BOW | 0.570 | 0.570 | 0.602 | 0.630 | 0.350 | 0.565 | 0.427 |
|  | GloVe | 0.533 | 0.620 | 0.549 | 0.593 | 0.572 | 0.553 | 0.610 |
|  | BERT-base | 0.629 | 0.602 | 0.517 | 0.523 | 0.400 | 0.552 | 0.657 |
|  | xlm-RoBERTa-base | 0.685 | 0.691 | 0.712 | 0.640 | 0.535 | 0.698 | 0.716 |
|  | RoBERTa-base | 0.676 | *0.753* | 0.665 | 0.651 | 0.509 | 0.697 | 0.696 |
|  | RoBERTa-large | 0.702 | 0.598 | 0.581 | 0.553 | 0.449 | 0.550 | 0.679 |
|  | PubMed-BERT-base | 0.561 | 0.651 | 0.604 | 0.624 | 0.520 | 0.612 | 0.629 |
|  | | | | | | | | |
| AUCROC | TF-IDF | 0.912 | *0.919* | 0.891 | 0.894 | 0.762 | 0.881 | 0.900 |
|  | BOW | 0.858 | 0.848 | 0.888 | 0.887 | 0.708 | 0.860 | 0.776 |
|  | GloVe | 0.862 | 0.889 | 0.858 | 0.882 | 0.790 | 0.873 | 0.897 |
|  | BERT-base | 0.900 | 0.900 | 0.832 | 0.837 | 0.772 | 0.867 | 0.905 |
|  | xlm-RoBERTa-base | 0.913 | 0.919 | 0.916 | 0.900 | 0.826 | 0.921 | 0.911 |
|  | RoBERTa-base | 0.916 | *0.938* | 0.918 | 0.906 | 0.798 | 0.925 | 0.922 |
|  | RoBERTa-large | 0.911 | 0.913 | 0.886 | 0.869 | 0.752 | 0.874 | 0.889 |
|  | PubMed-BERT-base | 0.899 | 0.910 | 0.877 | 0.866 | 0.785 | 0.904 | 0.896 |
| Note: The best performance for both static and contextual text representation trained ML classifiers is highlighted in italic. | | | | | | | | |
